# Incidence of COVID-19 in patients exposed to chloroquine and hydroxychloroquine: results from a population-based prospective cohort in Catalonia, Spain, 2020

**DOI:** 10.2807/1560-7917.ES.2021.26.9.2001202

**Published:** 2021-03-04

**Authors:** Rosa Maria Vivanco-Hidalgo, Israel Molina, Elisenda Martinez, Ramón Roman-Viñas, Adrián Sánchez-Montalvá, Joan Fibla, Caridad Pontes, César Velasco Muñoz, on behalf of the Real World Data Working Group

**Affiliations:** 1 Agència de Qualitat i Avaluació Sanitàries de Catalunya (AQuAS), Departament de Salut Generalitat de Catalunya, Carrer de Roc Boronat 81-95, Barcelona, Spain; 2 These authors contributed equally; 3 Infectious Diseases Department, Hospital Universitario Vall d'Hebron, PROSICS Barcelona, Universitat Autònoma de Barcelona, Barcelona, Spain; 4 Unitat de Genètica Humana, Departament de Ciències Mèdiques Bàsiques, Universitat de Lleida, Av. Alcalde Rovira Roure, 80, 25198, Lleida, Spain; 5 Institut de Recerca Biomedica de Lleida, Av. Alcalde Rovira Roure, 80, 25198, Lleida, Spain; 6 Gerència del Medicament – Àrea Assistencial, Servei Català de la Salut, Travessera de les Corts, 131-159, 08028 Barcelona, Spain; 7 Departament de Farmacologia, de Terapèutica i de Toxicologia, Universitat Autònoma de Barcelona, Av. de Can Domènech, 737, 08193 Cerdanyola del Vallès, Barcelona, Spain; 8 Members of the Real World Data Working Group have been acknowledged at the end of the article

**Keywords:** SARS-CoV-2, COVID-19, coronavirus, chloroquine, hydroxychloroquine, pre-exposure prophylaxis

## Abstract

**Background:**

Several clinical trials have assessed the protective potential of chloroquine and hydroxychloroquine. Chronic exposure to such drugs might lower the risk of infection with severe acute respiratory syndrome coronavirus 2 (SARS-CoV-2) or severe coronavirus disease (COVID-19).

**Aim:**

To assess COVID-19 incidence and risk of hospitalisation in a cohort of patients chronically taking chloroquine/hydroxychloroquine.

**Methods:**

We used linked health administration databases to follow a cohort of patients with chronic prescription of hydroxychloroquine/chloroquine and a control cohort matched by age, sex and primary care service area, between 1 January and 30 April 2020. COVID-19 cases were identified using International Classification of Diseases 10 codes.

**Results:**

We analysed a cohort of 6,746 patients (80% female) with active prescriptions for hydroxychloroquine/chloroquine, and 13,492 controls. During follow-up, there were 97 (1.4%) COVID-19 cases in the exposed cohort and 183 (1.4%) among controls. The incidence rate was very similar between the two groups (12.05 vs 11.35 cases/100,000 person-days). The exposed cohort was not at lower risk of infection compared with controls (hazard ratio (HR): 1.08; 95% confidence interval (CI): 0.83–1.44; p = 0.50). Forty cases (0.6%) were admitted to hospital in the exposed cohort and 50 (0.4%) in the control cohort, suggesting a higher hospitalisation rate in the former, though differences were not confirmed after adjustment (HR: 1·46; 95% CI: 0.91–2.34; p = 0.10).

**Conclusions:**

Patients chronically exposed to chloroquine/hydroxychloroquine did not differ in risk of COVID-19 nor hospitalisation, compared with controls. As controls were mainly female, findings might not be generalisable to a male population.

## Introduction

The coronavirus disease (COVID-19) outbreak that started in January in China evolved into a pandemic with global cumulative numbers totalling 110.7 million confirmed cases and over 2.4 million deaths by 23 February 2021, with figures constantly increasing [1].

Since the beginning of the pandemic, non-pharmaceutical interventions such as physical distancing, lockdowns and the use of face masks in public spaces have been implemented in an effort to reduce transmission of SARS-CoV-2. However, such strict non-pharmaceutical interventions can only be maintained for a limited time without critically affecting citizens’ psychological health and the economy.

Prophylactic pharmaceutical interventions for COVID-19 have been explored and developed since the outset of the pandemic. Early clinical reports from China and France suggested the potential antiviral activity of 4-aminoquinolines (chloroquine (CQ) and its analogue, hydroxychloroquine (HCQ)) against COVID-19 [2,3]. Supported by preclinical evidence of its activity in cell culture [4], both testing and empirically using these drugs in the prevention and treatment of COVID-19 increased [5]. However, a retrospective cohort study reported in July 2020 that continuous HCQ therapy does not prevent infection with SARS-CoV-2, based on a large healthcare database analysis in Israel [6].

CQ is one of the most frequently prescribed drugs in the world and has been used for treating malaria and as chemoprophylaxis in travellers for decades, even for long periods of exposure [7]. Its safety profile is well known and its risk-benefit is deemed favourable to treat and prevent malaria [8]. HCQ, a chloroquine hydroxylated derivate, has also been widely used. Data from observational studies suggest that HCQ is threefold less toxic than CQ; therefore, while the type of adverse events is similar for both drugs, they occur less frequently with HCQ [9]. Moreover, CQ and HCQ are known immunomodulators that have been used for decades as chronic treatments in certain rheumatological diseases [10-12].

Based on this background, several clinical trials were designed to assess the protective potential of 4-aminoquinolines (CQ or HCQ) against SARS-CoV-2 infection, given as pre-exposure and post-exposure prophylaxis in highly exposed populations, such as healthcare professionals (clinicaltrials.gov register: NCT04303507, NCT04308668, NCT04304053).

During the first wave of the pandemic it was therefore reasonable to consider whether people who are chronically taking CQ or HCQ for any therapeutic indication may have less risk of SARS-CoV-2 infection and, if they contract COVID-19, whether they develop less severe disease.

For this reason, we have compared the incidence of COVID-19 in people who are chronic users of CQ or HCQ and those who are not, among people living in Catalonia, Spain.

## Methods

Catalonia is an autonomous province in the west of Spain that has universal public healthcare coverage, including primary and hospital healthcare, as well as prescription drug costs. Integrated registries are used to manage administrative and clinical information on the entire population in Catalonia, i.e. 7.5 million people, including invoicing systems for pharmacies, episode diagnoses for budgeting and payment purposes, clinical information exchange systems across healthcare providers and dedicated registries for epidemiological surveillance.

We report this study according to the guidelines for observational studies using routinely collected data.

### Registry features and data acquisition

A Catalan central registry of insured persons allows linking of information at the individual level between all health administration databases, and includes individual sociodemographic data and aggregated primary care service area data. An individual’s healthcare identification code allows Catalan residents to be tracked across several health administration databases, including the acute and emergency hospitals and emergency discharge datasets, conjunt mínim bàsic de dades d'hospitalitzats d'aguts (CMBD-HA) and conjunt mínim bàsic de dades d’urgències hospitalàries (CMBD-URG); the pharmacy invoicing database; the episode diagnosis database for primary care, conjunt mínim de dades d'atenció primària (CMBD-AP), which records information on comorbidity and date of diagnosis; the laboratory database, including data of SARS-CoV-2 nucleic acid amplification test results; and the specific epidemiological mandatory registry for SARS-CoV-2 infection. All CMBD databases register the information using the Ninth and Tenth Revisions of the International Classification of Diseases codes (ICD-9 and ICD-10) [13,14], and the pharmacy invoicing database uses Anatomical Therapeutic Chemical/Defined Daily Dose index codes established by the World Health Organization Collaborating Centre in Oslo, Norway. The registries have an automated data validation system to check consistency of data and identify potential errors, and external audits are performed periodically to ensure quality and reliability of data.

### Study design and participants

We conducted a population-based, prospective cohort study using linked health administration databases in Catalonia, Spain. The first Catalan case in the COVID-19 outbreak was reported in late February, lockdown was declared on 13 March and peak incidence was reached in early April, with a progressive decline in incidence thereafter. Thus, we studied the period between 1 January and 30 April 2020.

The exposed cohort included patients who, on 1 January 2020, had been dispensed CQ/HCQ treatment in the last 6 months according to the pharmacy invoicing database. The Catalan central registry of insured persons was used to select a control cohort of people who, on 1 January 2020, did not have any invoices for CQ/HCQ in the same period. For each exposed patient, two non-exposed individuals (controls) of the same age, sex and primary care service area were chosen.

Information regarding baseline comorbidities was obtained from the CMBD-AP using the ICD-9 given in Box 1. A comorbidity index was also used (Adjusted Morbidity Groups), a recently developed morbidity measurement that enables classification of the population into five morbidity groups (very low risk, low risk, moderate risk, high risk, very high risk) [15]. Information regarding socioeconomic status was also retrieved from the Catalan central registry of insured persons for every subject using two levels (individual and primary care service area). Individual socioeconomic status was derived from the individual yearly income range information used to calculate pharmacy copayment. Copayment ranges are derived from tax declarations and/or social security benefits received, as follows: exempted (non-working population or people receiving non-contributory pension), < €18,000 ($US 20,468) income per year; €18,000 ($US 20,468) to €100,000 ($US 113,710) income per year; and > €100 000 ($US 113,710) income per year. The primary care service area socioeconomic status is defined by the index of deprivation that ranges from 0 (less deprived) to 100 (more deprived); this was also a control cohort matching parameter [16].

Box 1 **ICD-9 codes used for baseline comorbidities, Catalonia, Spain, 2020**

**Asthma:** 493xx

**Chronic obstructive pulmonary disease:** 491.0, 419.2x, 491.8, 491.9, 492xx, 494x, 496

**Chronic renal failure:** 403.01, 403.11, 403.91, 585xx, 586xx, 588.0

**Congestive heart failure:** 398.91, 402.01, 402.11, 402.91, 404.01, 404.03, 404.11, 404.13, 404.91, 404.93,428xx

**Diabetes mellitus:** 250xx

**Dementia:** 290xx, 331.0, 331.1, 331.2

**Hypertension**: 401x, 402xx, 403xx, 404xx, 405xx

**Ischaemic heart disease:** 410xx, 411xx, 412, 413xx, 414xx

**Liver cirrhosis:** 571.2, 571.5

**Malignant neoplasm:** 140xx-230xx

**Stroke:** 430, 431,432x, 433xx, 434xx, 436, 437, 437.0, 437.1,437.8, 437.9, 438xx

ICD-9: Ninth revision of the International Classification of Diseases.

### Estimation of chronic exposure to chloroquine/hydroxychloroquine

We considered that patients had chronic exposure to CQ/HCQ if—according to the pharmacy invoicing database—they had been dispensed CQ/HCQ (ATC P01BA01 or P01BA02) at least once in January 2020 and also during the previous 6 months or more, with this treatment ongoing during the study period.

### Outcome

Our main outcome of interest was incidence of SARS-CoV-2 infection or COVID-19 diagnosis, as defined by one or more of the following: (i) a record with ICD-10 codes B97.29 or B34.2, as retrieved from the CMBD-HA, CMBD-URG or CMBD-AP datasets; (ii) a record of infection retrieved from the specific registry for SARS-CoV-2 infection or (iii) a positive SARS-CoV-2 RT-PCR test in the laboratory database. Patients were followed from 1 January 2020 until diagnosis or until 30 April 2020 (end of follow-up). As a secondary outcome, we also retrieved data on admission to hospital due to COVID-19.

### Statistical analysis

We used descriptive statistics to show the baseline demographic and comorbidity information of the individuals included in our analyses. Incidence rate of SARS-CoV-2 infection was calculated for both the exposed cohort and the control cohort. The numerator of the incidence was the number of subjects with SARS-CoV-2 infection observed during follow‐up. The denominator, in person‐days, was the follow‐up duration, which ended on 30 April 2020, at the time of a SARS-CoV-2 infection diagnosis, on the date of death or at the last available follow-up.

For the time to event analyses for the main outcome (SARS-CoV-2 infection or COVID-19), cumulative incidence of event curves were estimated for each cohort (exposed and control), considered separately, using the Kaplan-Meier method and compared statistically using the log rank test. We fitted a priori multivariable Cox proportional hazards regression model adjusted for all comorbidities and individual socioeconomic status to determine which variables were associated with an increased risk of infection.

The same analysis was applied to time to event for the secondary outcome (admission to hospital due to COVID-19).

We checked the proportional hazards assumption for all covariates using graphical methods (inspection of log minus log plot of survival).

R statistical software, version R-4.0.0 was used for all analyses [17].

### Ethical statement

No patients were involved in setting the research question or the outcome measures, nor were they involved in the design or implementation of the study. Data from different health administration databases were linked and de-identified by a team not involved in the study analysis; only a full de-anonymised database was available to the study investigators. The study protocol was approved by the Ethical Review Board of Vall d’Hebron University Hospital (Code VHI-HCQ-2020–01), Barcelona, Spain.

## Results

At the start of the follow-up (1 January 2020), 6,746 patients were identified as chronic users of CQ (114)/HCQ (6,648) and matched to 13,492 controls. All but one patient had at least one additional dispensation of CQ/HCQ during the study period. Baseline characteristics are described in Table 1. Median age in both cohorts was 57 years (interquartile range: 45–69) and 84.2% were women. In general, patients in the exposed cohort had more comorbidities and lower individual socioeconomic status than those in the control cohort.

**Table 1:** Characteristics of control cohort and cohort exposed to chloroquine/hydroxychloroquine, Catalonia, Spain, 1 January 2020

Characteristics	Control (n = 13,492)		Exposed (n = 6,746)		Total (N = 20,238)		p value
	n	%	n	%	N	%	
**Age (years)**							
Mean (SD)	57.1 (15.8)	–	57.1 (15.8)	–	57.1 (15.8)	–	1.000
Median (Q1, Q3)	57.0 (45.0, 69.0)	–	57.0 (45.0, 69.0)	–	57.0 (45.0, 69.0)	–	
Min–Max	8.0–96.0	–	8.0–96.0	–	8.0–96.0	–	
**Sex**							
Female	11,366	84.2	5,683	84.2	17,049	84.2	1.000
Male	2,126	15.8	1,063	15.8	3,189	15.8	
**Comorbidities**							
Hypertension	4,056	30.3	2,513	37.3	6,569	32.7	< 0.001
Diabetes	1,512	11.2	763	11.3	2,275	11.3	0.841
Congestive heart failure	494	3.7	415	6.2	909	4.5	< 0.001
COPD	896	6.7	829	12.3	1,725	8.5	< 0.001
Chronic renal disease	798	5.9	851	12.6	1,649	8.2	< 0.001
Asthma	954	7.1	633	9.4	1,587	7.9	< 0.001
Dementia	227	1.7	78	1.2	305	1.5	0.004
Liver cirrhosis	63	0.5	60	0.9	123	0.6	< 0.001
Stroke	629	4.7	444	6.6	1,073	5.3	< 0.001
Coronary artery disease	517	3.8	409	6.1	926	4.6	< 0.001
Cancer	1,530	11.4	880	13.1	2,410	11.9	< 0.001
**Tobacco use**							
Non-smoker	5,319	58.7	2,756	56.1	8,075	57.8	< 0.001
Current smoker	2,310	25.5	1,238	25.2	3,548	25.4	
Former smoker	1,440	15.9	919	18.7	2,359	16.9	
**AMG**							
Very low risk	8,238	61.2	1,817	27.0	10,055	49.8	< 0.001
Low risk	3,352	24.9	2,753	40.9	6,105	30.2	
Moderate risk	1,442	10.7	1,581	23.5	3023	15.0	
High risk	339	2.5	467	6.9	806	4.0	
Very high risk	82	0.6	114	1.7	196	1.0	
**IS level**							
Exempts	540	4.0	325	4.8	865	4.3	< 0.0012
< 18,000 €	8,056	59.7	4,170	61.8	12,226	60.4	
18,001–100,000 €	4,776	35.4	2,193	32.5	6,969	34.4	
> 100,000 €	120	0.9	58	0.9	178	0.9	
**PCL deprivation index**							
0–32.2	3,490	26.3	1,745	26.3	5,235	26.3	1.000
32.2–41.2	3,210	24.2	1,605	24.2	4,815	24.2	
41.2–49.7	3,296	24.8	1,648	24.8	4,944	24.8	
49.7–100	3282	24.7	1,641	24.7	4,923	24.7	
**CQ/HCQ**	**0**	**0**	**6,746**	**100.0**	**6,746**	**33.3**	< 0.001
CQ	0	0	114	1.7	114	1.7	
HCQ	0	0	6,648	98.5	6,648	98.5	
**Hospitalisation**	**50**	**0.4**	**40**	**0.6**	**90**	**0.4**	**0.025**
**COVID-19 diagnosis**	**183**	**1.4**	**97**	**1.4**	**280**	**1.4**	**0.640**

AMG: Adjusted Morbidity Groups; COPD: chronic obstructive pulmonary disease; COVID-19: coronavirus disease; CQ: chloroquine; HQ: hydroxychloroquine; IS level: individual socioeconomic level; PCL deprivation index: index of deprivation at primary care service area level; Q1: first quartile; Q3: third quartile; SD: standard deviation.

**Figure 1:**
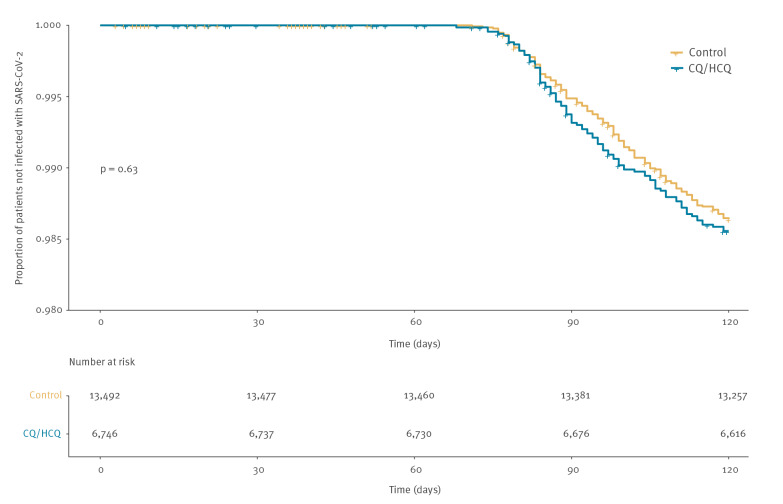
Cumulative incidence of SARS-CoV-2 infection in the cohort exposed to chloroquine/hydroxychloroquine and the control cohort, Catalonia, Spain, 1 January–30 April 2020

A total of 97 cases in the exposed cohort (1.4%) and 183 (1.4%) in the control cohort had SARS-CoV-2 infection during the follow-up. The incidence rate was similar between both cohorts (12.05 vs 11.35 cases/100,000 person-days). No differences were observed in the crude cumulative incidence of event (SARS-CoV-2 infection) curves (Figure 1).

Control: non-exposed control cohort; CQ/HCQ: cohort exposed to chloroquine/hydroxychloroquine; SARS-CoV-2: severe acute respiratory syndrome coronavirus 2.

Table 2 shows hazard ratios (HRs) for time-to-infection for the CQ/HCQ cohort after adjusting for comorbidities and individual socioeconomic status. Patients chronically exposed to CQ/HCQ were not at lower risk of infection compared to controls (HR: 1.08; 95% confidence interval (CI): 0.83–1.44; p = 0.50).

**Table 2:** Adjusted hazard ratios and 95% confidence intervals for episodes of SARS-CoV-2 infection by comorbidities and individual socioeconomic status, Catalonia, Spain, 30 April 2020

Characteristics	Adjusted hazard ratio	Lower 0.95 CI	Upper 0.95 CI	p value
Hypertension	0.93	0.68	1.27	0.65
Diabetes	1.17	0.81	1.70	0.40
Heart failure	1.24	0.76	2.04	0.40
COPD	1.59	1.08	2.35	0.01
Chronic renal disease	1.30	0.87	1.95	0.20
Asthma	1.19	0.78	1.82	0.40
Dementia	4.33	2.61	7.17	< 0.001
Liver cirrhosis	0.70	0.10	5.03	0.72
Stroke	1.38	0.88	2.16	0.16
Coronary artery disease	1.27	0.77	2.10	0.35
Cancer	1.05	0.73	1.52	0.79
Current smoker	0.67	0.46	0.93	0.03
Former smoker	1.32	0.95	1.82	0.10
IS level < 18,000 €	1.04	0.55	1.99	0.90
IS level 18,001–100,000 €	0.85	0.44	1.68	0.60
IS level > 100,000 €	0.79	0.10	6.17	0.80
CQ/HCQ exposure	1.08	0.83	1.44	0.50

CI: confidence interval; COPD: chronic obstructive pulmonary disease; CQ/HCQ: chloroquine/hydroxychloroquine; IS level: individual socioeconomic level; SARS-CoV-2: severe acute respiratory syndrome coronavirus 2.

**Figure 2:**
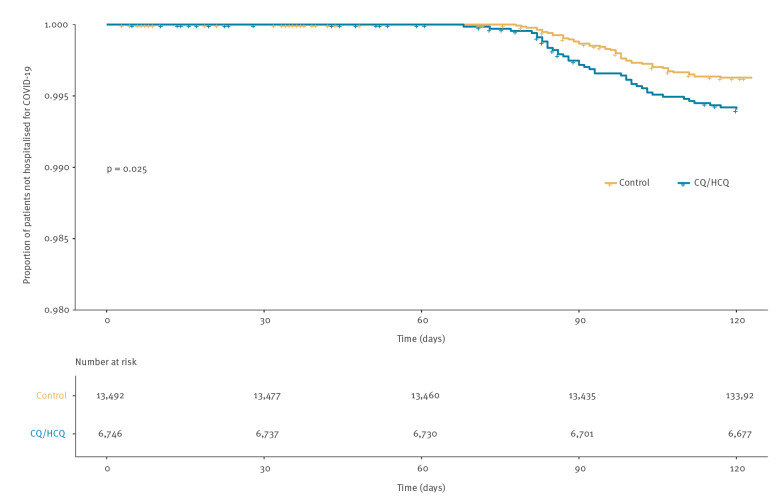
Cumulative incidence of hospitalisation for COVID-19 in cohort exposed to chloroquine/hydroxychloroquine and control cohort, Catalonia, Spain, 30 April 2020

Unadjusted estimates for risk of hospitalisation for COVID-19 showed that 40 (0.6%) cases were admitted to public hospitals in the exposed cohort and 50 (0.4%) in the control cohort, with significant differences between the two cohorts in the unadjusted cumulative incidence of event curves (Figure 2).

Control: non-exposed control cohort; COVID-19: coronavirus disease: CQ/HCQ: cohort exposed to chloroquine/hydroxychloroquine.

Adjusted estimates by comorbidities and socioeconomic status did not show significant differences (HR: 1.46; 95% CI: 0.91–2.34; p = 0.10). In particular, dementia was associated with a substantial increase of risk (Table 3).

**Table 3:** Adjusted hazard ratios and 95% confidence intervals for hospitalisation for COVID-19, by comorbidities and individual socioeconomic status, Catalonia, Spain, 30 April 2020

Characteristics	Adjusted hazard ratio	Lower 0.95 CI	Upper 0.95 CI	p value
Hypertension	1.40	0.82	2.38	0.20
Diabetes	1.04	0.54	1.97	0.90
Heart failure	0.83	0.33	2.09	0.70
COPD	1.17	0.57	2.40	0.60
Chronic renal disease	1.70	0.89	3.25	0.10
Asthma	1.42	0.69	2.90	0.30
Dementia	1.17	0.28	4.90	0.80
Liver cirrhosis	2.01	0.27	14.72	0.50
Stroke	1.28	0.57	2.88	0.50
Coronary artery disease	0.89	0.34	2.33	0.80
Cancer	1.44	0.80	2.60	0.20
Current smoker	0.58	0.29	1.16	0.10
Former smoker	1.23	0.68	2.19	0.48
IS level < 18,000 €	1.91	0.46	7.83	0.37
IS level 18,001–100,000 €	1.08	0.25	4.76	0.9
IS level > 100,000 €	0	0	–	0.99
CQ/HCQ exposure	1.46	0.91	2.34	0.10

CI: confidence interval; COPD: chronic obstructive pulmonary disease; COVID-19: coronavirus disease; CQ/HCQ: chloroquine/hydroxychloroquine; IS level: individual socioeconomical level.

## Discussion

After comparing the two cohorts, we found no differences between the incidence of COVID-19 in those under long-term CQ/HCQ therapy and the controls. When analysing unadjusted risk of hospitalisation for COVID-19, patients receiving 4-aminoquinolines had more risk of hospitalisation; however, this effect did not persist after adjustment for comorbidities, suggesting that it might be because of the higher proportion of comorbidities among the exposed patients.

The rapid spread of SARS-CoV-2 led to the need to seek pharmaceutical prevention strategies while waiting for an effective and safe vaccine. Different strategies have been evaluated, mainly in high-risk groups such as healthcare workers. CQ/HCQ were the first candidates to be evaluated, because of evidence shown in experimental models and incipient observational studies. As at March 2020, more than 80 clinical trials have been registered to assess the potential activity of CQ/HCQ against COVID-19, including the full spectrum of symptoms. Although early observational studies reported positive effects, as larger observational studies and clinical trials emerged, the evidence to support the use of 4-aminoquinolines declined. There are already several clinical trials that have failed to demonstrate clinical or virological efficacy of these drugs [18,19], while others concluded that their use could even be harmful [20,21].

The current evidence from clinical trials refers to the use of 4-aminoquinolines as either a treatment of established infection or prophylaxys after exposure. Results from clinical trials showed that after high-risk or moderate-risk exposure to SARS-CoV-2, HCQ did not prevent illness compatible with COVID-19 or confirmed COVID-19 when used as postexposure prophylaxis within 4 days after exposure [22,23].

The observed incidence rate alone is not an appropriate measure of the protective efficacy or harmful effects of pharmaceutical interventions against SARS-CoV-2, since despite comorbidity adjustments, colliding bias and residual confounding may persist. Antiviral activity of either CQ/HCQ could still have a role in reducing population transmission of SARS-CoV-2 or reducing the severity of COVID-19 in individuals before they are exposed. This should be studied through randomised, prospective comparisons [24]. Therefore, although the observed incidence rate in patients exposed to CQ/HCQ could be the same as in controls, as in our study, it would still be reasonable to accept a strategy that may reduce the viral load and diminish symptoms and severity in infected individuals.

We observed that patients receiving CQ/HCQ had more admissions to hospital than controls (0.6% vs 0.4%, p = 0.025), but the adjusted estimates by comorbidities did not show significant differences in the risk of hospitalisation (HR: 1.5 95% CI: 0.9–2.3; p = 0.10), suggesting that comorbidity burden could explain the differences. In particular, although comorbidities were considered as covariates to adjust the risk models in our analysis, dementia showed a significant association with higher risk of infection. The greatest proportion of patients affected by COVID-19 has been in long-term care facilities, with nursing home residents accounting for ca 25–50% of documented deaths [25,26]. As dementia patients are likely to live in nursing homes, this association could also be attributed to living situation; regardless, we might still consider dementia as a surrogate marker.

We did not find individual socioeconomic status to have any impact on risk of infection; it was only associated with the risk of hospital admission for COVID-19. However, reports in other countries have shown that the most disadvantaged and vulnerable people are at increased risk for contracting COVID-19, because of possible factors such as poorer baseline health status, closer social contacts and lack of access to healthcare [27,28]. In our scenario, the universal healthcare system (RDL 7/2018) [29] in Spain might have played a role in our results, as access to healthcare would not have been limited regardless of socioeconomic status.

Limitations of the available data and study approach must also be acknowledged. We did not explore concurrent medications; some of the treatments used for autoimmune diseases may also influence both susceptibility to infection and severity of disease, and this may have led to residual confounding. We did not have information regarding the daily doses of CQ/HCQ per patient, and the dose could also influence the results. However, as this study analyses the effect of pre-exposure, we consider that chronic administration of higher doses than are commonly prescribed would not be feasible in general practice because of the expected side effects. As medication data were obtained from the pharmacy invoicing database, we could not assure treatment compliance. However, as these are mainly chronic treatments, we inferred compliance by selecting those individuals with chronic dispensation for 6 months before starting follow-up. The risk of SARS-CoV-2 infection is also influenced by environmental characteristics, which have not been controlled. To overcome this, primary care service area was also a matching parameter for the control cohort. Regarding hospitalisation, episodes of inpatient and outpatient care carried out in private hospitals were not available for this analysis. However, during the COVID-19 epidemic all private hospitals reported COVID-19 cases to the public healthcare system, so we can be confident that all cases were identified. Finally, around 80% of the study population were female, probably because current prescriptions for CQ/HCQ are for autoimmune diseases (such as rheumatoid arthritis), which are known to be more common in women. This is a major limitation with respect to generalisation of the results to a male population.

Clinical trials continue to be the gold standard to generate evidence regarding drug efficacy, but they are difficult to implement in rapidly evolving, emergency conditions, because of demanding implementation requirements. However, this analysis of the impact of long-term 4-aminoquinolines therapy on the incidence of COVID-19 is intended to serve as supportive information to guide clinical practice while more robust evidence is generated.

### Conclusions

Patients chronically exposed to 4-aminoquinolines (CQ/HCQ) did not differ from non-exposed controls with regard to risk of infection by SARS-CoV-2. Although patients receiving these drugs were hospitalised more often, the risk may not be attributed to the drug, but to a higher proportion of comorbidities.
